# Dementia risk factor assessment in a local Alzheimer’s prevention population: a German cross-sectional, observational study

**DOI:** 10.1016/j.tjpad.2026.100556

**Published:** 2026-04-03

**Authors:** Lena Sannemann, Michelle Gerards, Lara Bohr, Frederic Brosseron, Claus Escher, Franziska Kalthegener, Theresa Müller, Alfredo Ramírez, Philip Zeyen, Frank Jessen, Ayda Rostamzadeh

**Affiliations:** a Department of Psychiatry and Psychotherapy, Faculty of Medicine and Cologne University Hospital, University of Cologne, Kerpener Str. 62, 50937 Cologne, Germany; b German Center for Neurodegenerative Diseases (DZNE), Venusberg-Campus 1/99 53127 Bonn, Germany; c Department of Geriatric Psychiatry and Cognitive Disorders, University Hospital Bonn, Venusberg-Campus 1 53127 Bonn, Germany; d Department of Pediatrics, Faculty of Medicine and Cologne University Hospital, University of Cologne, Kerpener Str. 62, 50937 Cologne, Germany; e Cluster of Excellence Cellular Stress Responses in Aging-associated Diseases (CECAD), Faculty of Medicine and Cologne University Hospital, University of Cologne, Joseph-Stelzmann-Straße 26 50931 Cologne, Germany

**Keywords:** Alzheimer’s disease, Dementia, Prevention, Risk assessment, Risk communication, Modifiable risk factors

## Abstract

**Background:**

The risk for dementia is to a significant extent driven by potentially modifiable factors. Prevention strategies are increasingly aiming at individually tailored risk reduction approaches, particularly in light of emerging Brain Health Services for dementia prevention (dBHS).

**Methods:**

The cross-sectional observational study “Individual Risk Profiling for Alzheimer's and Dementia Prevention” (INSPIRATION) assessed the individual risk factors of 162 participants of the local Cologne Alzheimer Prevention Registry and provided individual feedback on risk profiles during a single visit. We analysed the frequency and patterns of risk factors and explored their association with cognition and Alzheimer’s disease (AD) plasma biomarkers.

**Findings:**

The most common risk factors in this population were obesity, non-adherence to a Mediterranean diet, low subjective sleep quality, subjective experience of stress, and hearing impairment. A principal component analysis (PCA) revealed six principal components (PC), which we labeled as (1) psychosocial factors, (2) blood pressure, (3) physical condition, (4) hearing impairment, (5) lifestyle, and (6) substance use. We found isolated associations between PCs, cognition, and AD plasma biomarkers.

**Interpretation:**

These findings provide initial insights into which risk factors may be most relevant and actionable for highly-educated and prevention-motivated populations likely to seek dBHS. Interventions addressing the domains of psychosocial factors, physical condition, and lifestyle may be particularly relevant to consider for a personally tailored risk reduction approach in comparable populations.

**Funding:**

The study was funded by research funds of the Medical Faculty and the University Hospital Cologne, University of Cologne and the non-profit association Kölner Verein für seelische Gesundheit e.V.

**ABBREVIATIONS**
AD: Alzheimer’s disease
APOE: Apolipoprotein E
Aß: Amyloid beta
BMI: Body mass index
BRS: Brief Resilience Scale
CERAD: Consortium to Establish a Registry for Alzheimer’s Disease
CSF: Cerebrospinal fluid
CV: coefficient of variation
HADS: Hospital Anxiety and Depression Scale
KMO: Kaiser-Meyer-Olkin measure
LSNS: Lubben Social Network Scale
MCI: Mild cognitive impairment
MEDAS: Mediterranean Diet Adherence Screener
PASE: Physical Activity Scale for the Elderly
PC: Principal component
PCA: Principal component analysis
p-tau: Phosphorylated tau
SCD: Subjective cognitive decline
SCD-I: Subjective cognitive decline – Interview
SNP: Single-nucleotide polymorphisms
SPSS: Statistical Package for the Social Sciences – 28th edition
WVT: Whispered voice test

## 1 Introduction

Epidemiological data suggest that up to 45 % of dementia risk is related to potentially modifiable factors [1]. This estimate has contributed to worldwide awareness and campaigns for dementia risk reduction [2]. A wide range of clinical trials with single or multicomponent risk reduction interventions have partially, but not consistently, shown a beneficial effect on cognition or a slowing of cognitive decline in individuals at either increased risk for dementia or in unselected volunteers [3]. To improve the efficacy of risk reduction interventions, the idea of personalised dementia risk reduction approaches, as opposed to generic multicomponent strategies, has emerged [4]. Personalised dementia prevention is also the concept of the recently proposed Brain Health Services for dementia prevention (dBHS), which are designed for cognitively unimpaired individuals who seek medical help, including those with purely subjective cognitive decline (SCD) or with increased worries about developing dementia. dBHS aim to provide individual risk factor assessment, risk communication, individual risk reduction strategies, and cognitive enhancement [5]. Currently, dBHS are in the development phase and have not yet been implemented on a larger scale in healthcare.

To establish personalised dementia prevention strategies and prepare the development of a local dBHS, we founded the Cologne Alzheimer Prevention Center (Kölner Alzheimer Präventionszentrum, KAP) [6]. Building on this infrastructure, we conducted the *Individual Risk Profiling for Alzheimer's and Dementia Prevention* (INSPIRATION) study, which aimed to examine the distribution of dementia-related risk factors and gain experience with a one-step counselling approach in a population likely to seek dBHS. We further explored associations between risk factors and cognition, apolipoprotein E (APOE) status, and plasma biomarkers. The proceedings of INSPIRATION may help inform the establishment of a dBHS in comparable populations.

## 2 Methods

The INSPIRATION study was designed as an exploratory, cross-sectional observational study to assess profiles of established dementia risk factors and provide individualised counselling in a population motivated to engage in dementia prevention.

### 2.1 Participants and recruitment

All participants were recruited through the KAP’s Cologne Alzheimer’s Prevention Registry, which is advertised to the general population in the city of Cologne, Germany, and is free of charge. Registry members receive quarterly newsletters on brain health and dementia topics via email, as well as updates on initiatives and studies related to the KAP. At the time of recruitment, the registry comprised 1198 individuals (63 % female) with a mean age of 64·3 [20–95] years.

The INSPIRATION study was announced via a single email as an initiative offering individual dementia risk assessment and risk reduction advice. Eligibility criteria included an age ≥ 50 years, while a dementia diagnosis was an exclusion criterion. The study recruitment period was from September 2020 to January 2022. Since the study aimed to explore the frequency and distribution of risk factors in the target population, the sample size was not restricted. The study consisted of a single on-site visit, which lasted approximately two hours and included risk assessment, risk communication, and standardised advice individually tailored to identified risk factors. In preparation for the study visit, participants were sent questionnaires about their medical history and lifestyle, which were reviewed on-site.

The study was conducted in accordance with the Declaration of Helsinki and the International Conference on Harmonization Good Clinical Practice guidelines, and the ethical committee of the Medical Faculty of the University of Cologne approved the study (ID:20–1096). All participants provided written informed consent prior to the study. The protocol was registered in the German Clinical Study Register (Deutsches Register Klinischer Studien, DRKS, ID:DRKS00020767).

### 2.2 Data collection and risk assessment

The assessments included demographics, medical and family history, questionnaires on modifiable risk factors, physical examination, cognitive testing, blood biomarker analysis, and APOE genotyping. Detailed information on the assessment of medical history and medication is available in Supplement S1. An overview of all variables, methods of assessments, and the cut-offs used to define the presence or absence of a risk factor is available in Supplement Table S1. To examine the prevalence of risk factors in this population, we focused on the modifiable factors described in the WHO Guidelines on risk reduction of cognitive decline and dementia [2], and by Livingston et al. 2020 [7]. To achieve comprehensive phenotyping of our population, this panel was expanded with further potentially modifiable factors that have been discussed in the literature, but had insufficient evidence to support inclusion in the work by the Lancet Commission [2,[7], [8], [9]].

#### 2.2.1 Lifestyle, physical, and mental health risk factors

The final risk factor panel comprised lifestyle factors such as adherence to the Mediterranean diet, physical activity, size of social network, alcohol and tobacco use, and participation in sports with a high risk of head injuries. It further included aspects of physical and mental health such as sleep quality, stress, resilience, loneliness, anxiety, depression, subjective hearing deficits, periodontal risk, and self-rated physical fitness. These risk factors were assessed using standardised questionnaires, and established cut-offs were applied to define their presence or absence (Supplement Table S1).

#### 2.2.2 Cognitive and psychiatric assessment

SCD was assessed using the SCD-Interview [10]. All participants were tested with the Consortium to Establish a Registry for Alzheimer’s Disease (CERAD+) neuropsychological test battery [11]. We used the publicly available age-, sex- and education-adjusted normative database for the CERAD (www.memoryclinic.ch). The global score of the CERAD battery according to Chandler et al. was calculated for every participant [12]. In addition, a memory score was calculated as the mean of the z-standardised scores of the word list learning, recall, recognition, and figure recall test. Performance below −1·5 standard deviation (SD) on one CERAD+ test or below −1·0 SD on two tests within the same cognitive domain or below −1·0 SD on three or more tests in three different cognitive domains [13,14] defined mild cognitive impairment (MCI). Furthermore, symptoms of depression and anxiety were assessed.

#### 2.2.3 Physical examination

Height and weight were measured to calculate the body mass index (BMI). Abdominal obesity was assessed by the waist-hip ratio. Blood pressure was measured, and hearing was tested with the Whispered Voice Test (WVT) [15].

### 2.3 Genotyping and Alzheimer’s disease biomarkers

Results from APOE genotyping and Alzheimer’s disease (AD) biomarkers were not included in the risk factor assessment itself, but were used to exploratively examine associations between risk factors and genetic or biological markers. The APOE status was determined by genotyping using two single-nucleotide polymorphisms (SNP), rs429358 and rs7412, defining the ε2, ε3, and ε4 alleles in the APOE gene by a TaqMan Allelic Discrimination Assay for each SNP (Thermo Fisher Scientific, Waltham, Massachusetts, United States) on a QuantStudio™ 6 Flex Real-Time PCR System.

Concentrations of Aβ40, Aβ42, GFAP, NFL, and p-tau-181 were measured using commercially available SIMOA® assays (Quanterix®, Billerica, USA, 103,670 Neurology 4-plex E and 103,414 p-tau-181 Advantage V2 assay kits, respectively). Samples and calibrators were measured in duplicates with a maximum accepted coefficient of variation (CV) of 20 % for samples. Samples exceeding this threshold were repeated, and samples with intrinsic high variance on repetition were omitted from analysis. In addition to the single biomarkers, the ratio Aβ42/Aβ40 and Aβ42/p-tau181 were calculated. Biomarker measurements were performed at the German Center for Neurodegenerative Diseases (DZNE) in Bonn.

### 2.4 Risk communication

While the risk assessment covered a large number of potential risk factors, risk communication was restricted to a selection of factors with robust evidence for effects on dementia risk, for which recommendations for reduction are available [1,2,7]. The risk factors communicated by the clinician (psychiatrist) included cognitive activity, physical activity, smoking, alcohol consumption, obesity, hypertension, depression, hearing loss, diet, stress, and sleep quality. The results of the blood biomarkers were not disclosed, the genetic test results were provided upon request. In this study, satisfaction with risk communication was not assessed. After six months, a follow-up was carried out via questionnaire inquiring about lifestyle changes since the study participation.

### 2.5 Statistical analysis

To describe the frequency and distribution of individual risk factors, we used means, standard deviations, and percentages. To explore risk factor patterns, we first applied a principal components analysis (PCA) with Varimax orthogonal rotation to the data to generate principal components (PC) of correlated risk factors. In accordance with PCA assumptions, we included only risk factors measured on a continuous scale. To improve interpretability, the scores for the Brief Resilience Scale (BRS) [16], Whispered Voice Test [17], the Physical Activity Scale for the Elderly (PASE) [18], the Lubben Social Network Scale (LSNS) [19], and the Mediterranean Diet Adherence Screener (MEDAS) [20] were inverted so that for all variables included in the PCA, a higher score indicated higher risk. The Kaiser-Meyer-Olkin (KMO) measure of sampling adequacy was 0·66, which is above the recommended cut-off. Bartlett's test of Sphericity was highly significant (*p*<·001), indicating that correlations between instruments were sufficiently large for a PCA. We considered PCs with eigenvalues greater than 1·0 as relevant. To define how many PCs should be kept in the analysis, the scree plot was reviewed. Each PC is represented as a combination of the original variables. Principal components were ordered by decreasing explained variance, with the first principal component (PC1) explaining the largest proportion of total variance.

To summarise individual results, a composite score (PC composite score) for each component was calculated. This was achieved by multiplying each original measure by its weight, which was derived from the PCA, and summing these for each PC, creating a composite score for each PC of each individual. These composite scores were then split into tertiles. A PC composite score in the highest tertile was classified as ‘high risk’ and in the low and middle tertile as ‘low to medium risk’. We used an UpSet plot to depict the distribution across participants [21].

Additionally, we explored associations between risk factors, cognition, AD biomarkers, and APOE genotype. Group differences in PC composite scores between APOE-4 carriers and non-carriers were calculated using Mann-Whitney U tests. Spearman's rank correlations were calculated to assess the associations between the PC composite scores, cognitive performance, and AD biomarkers. The correlation analyses of the AD biomarkers were corrected for age, sex and APOE carrier status. To explore age-related effects, analyses were stratified by age groups based on a median split. Bonferroni correction was applied to adjust for multiple comparisons.

Analyses excluded participants with missing data. A value *p* < 0·05 was considered significant in all statistical analyses. The analyses were carried out with IBM SPSS Statistics 28·0 (IBM Corp.,155 Armonk, NY) for Windows, JASP Team (2024, JASP Version 0.19.0), and UpSetR [21].

## 3 Results

### 3.1 Sample characteristics

A total of 162 participants were enrolled (63 % female) after a single email announcement. The mean age was 64·6 years (*SD*=8·1, [50–86]), and the mean years of education were 16·3. The distribution of APOE-4 carriers (31·6 %) corresponded to that of the general population, without evident enrichment for APOE-4 [22]. SCD was reported in 79·6 % of participants, and 48 individuals (29·6 %) fulfilled the MCI criteria. Additional demographic and medical characteristics, genetic and biomarker information, as well as cognitive test scores, are listed in Table 1.

**Table 1:** Demographic and medical characteristics of the INSPIRATION sample (*n* = 162).

	Variable	*M (SD)*
Demographics	Age	64·6 (8·1)
	Years of education	16·3 (2·6)
		
		*N* ( %)
	Sex (female)	102 (63·0)
	Retired	80 (49·4)
	Full or part-time employment	69 (42·6)
	Single household	30 (24·7)
	Family history of dementia	113 (69·8)
Physical examination	Systolic blood pressure ≥140 mmHg	106 (65·4)
	Overweight (BMI 25–29·9 kg/m^2^)	52 (32·1)
	Obesity (BMI ≥30 kg/m^2^)	28 (17·3)
	Hearing impairment (WVT)	50 (30·8)
APOE- ε4 status	APOE ε2/ε3	21 (13·0)
	APOE ε2/ε4	1 (0·6)
	APOE ε3/ε3	87 (53·7)
	APOE ε3/ε4	47 (29·0)
	APOE ε4/ε4	2 (1·2)
	Not determined	4 (2·5)
		*M (SD)*
Cognition	MMSE	29·5 (0·8)
	Chandler Score	89·38 (6·91)
	Memory Score	0·27 (0·63)
Plasma biomarkers	Aß40, pg/ml	99·24 (15·12)
	Aß42, pg/ml	7·55 (1·75)
	p-tau181, pg/ml	119·08 (59·03)
	Aß42/40, pg/ml	0·08 (0·01)
	p-tau181/Aß42, pg/ml	0·30 (0·18)
	NFL, pg/ml	16·49 (8·94)
	GFAP, pg/ml	119·08 (59·03)

Note. Plasma biomarker data with a variance coefficient > 20 % were excluded in quality control, leading to a sample size of *n* = 160 for p-tau181 and *n* = 159 for all other biomarkers.

APOE apolipoprotein E; Aß40 amyloid ß1–42; Aß42 amyloid ß1–42; BMI body mass index; GFAP glial fibrillary acidic protein; MMSE Mini-Mental State Examination; NFL neurofilament light chain; p-tau181 phospho-tau181; WVT Whispered Voice Test.

### 3.2 Risk factor frequency and distribution

#### 3.2.1 Frequency of modifiable risk factors

The most common risk factors were obesity, non-adherence to a Mediterranean diet, low subjective sleep quality, subjective experience of stress, and hearing impairment. The most frequent risk factors from medical history were hypercholesterolemia, hypertension, vitamin deficiency, hearing impairment, and a history of depression. Table 2 presents the prevalence of modifiable risk factors in this sample.

**Table 2:** Prevalence of modifiable medical and lifestyle risk factors in the INSPIRATION sample (*n* = 162).

	Risk factor	*N* ( %)
Medical history	Hypercholesterolemia	81 (50·0)
	Thyroid disease	59 (36·4)^a^
	Hypertension	55 (34·0)
	Hearing impairment	43 (26·5)
	Depression	40 (24·7)^a^
	Visual impairment	29 (17·9)
	Traumatic brain injury	18 (11·1)
	Vitamin B deficiency	13 (8·0)
	Diabetes mellitus type 2	13 (8·0)
	Anxiety	20 (6·2)
	Kidney disease	9 (5·6)
	Coronary heart disease	8 (4·9)
	Atrial fibrillation	8 (4·9)
	Olfactory impairment	7 (4·3)
	Stroke	4 (2·5)
	Parkinson’s disease	1 (0·6)
Medication	Antidepressant medication	36 (22·2)
	Proton-pump inhibitors	35 (21·6)
	Hormone replacement therapy	25 (15·4)
	Opioids	14 (8·6)
	Benzodiazepines	2 (1·2)
Lifestyle risk factors	Abdominal obesity (WHR)	120 (74·1)
	Low adherence to Mediterranean diet	113 (69·8)
	Bad quality of sleep	85 (52·5)
	Elevated stress	77 (47·5)
	Subjective hearing impairment	75 (46·3)
	Low physical activity	57 (35·2)^b^
	Elevated risk of periodontitis	55 (34·0)
	Objective hearing impairment (diagnosed)	43 (26·5)
	Low stress resilience	37 (22·8)
	Symptoms of anxiety (HADS-A)	26 (16·0)
	Subjective feeling of loneliness	20 (12·3)
	Elevated alcohol consumption	19 (11·7)
	Symptoms of depression (HADS-D)	13 (8·0)
	Sports with a high risk of head injury	11 (6·8)
	Small social network	10 (6·2)
	Tobacco consumption/smoking	8 (4·9)
	Symptoms of depression (GDS)	9 (5·6)

GDS, Geriatric Depression Scale; HADS-A, Hospital Anxiety and Depression Scale – Subscale Anxiety; HADS-D, Hospital Anxiety and Depression Scale – Subscale Depression; WHR, waist-hip-ratio.

a n = 161.

b n=160.

#### 3.2.2 Principal component analysis

**Fig. 1:**
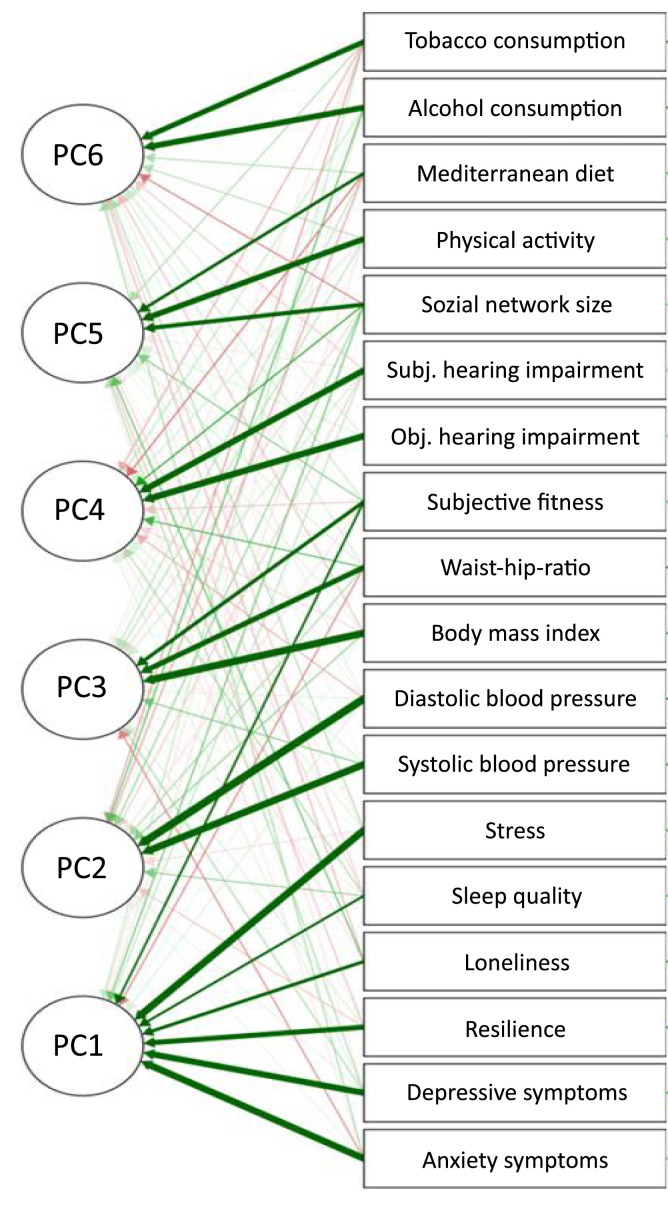
Path diagram of principal component analysis (PCA). Path diagram displaying the composition of each of the following principal components (PC) after Varimax orthogonal rotation: PC1, psychosocial factors; PC2, blood pressure; PC3, physical condition; PC4, hearing impairment; PC5, lifestyle; PC6, substance use.

The exploratory PCA yielded a six-component solution that explained 61·82 % of the total variance. The PCs were labeled as (1) psychosocial factors, (2) blood pressure, (3) physical condition, (4) hearing impairment, (5) lifestyle, and (6) substance use, with psychosocial factors explaining the highest variance in our sample. Table 3 presents the included variables, the component loadings, and commonalities of the rotated solution. The first component loaded most on stress, resilience, symptoms of depression and anxiety, loneliness, and sleep. The second component loaded on systolic and diastolic blood pressure. The third component loaded on BMI, waist-hip ratio, and subjective assessment of physical fitness. The fourth component loaded on subjective and objective hearing impairment. The fifth component loaded on the pattern of physical inactivity, social network size, and non-adherence to the Mediterranean diet. The sixth component loaded on alcohol and tobacco consumption. The path diagram and component solution are shown in Fig. 1.

**Table 3:** Rotated structure matrix for PCA with varimax rotation.

	Rotated Component Coefficients					
	Psychosocial Factors	High Blood Pressure	Poor physical constitution	Hearing impairment	Unhealthy lifestyle	Drug consumption
Stress (PSS total score)	**0·828**					
Anxiety symptoms (HADS anxiety subscore)	**0·822**					
Depessive symptoms (HADS depression subscore)	**0·791**					
Stress resilience (BRS total score)	**0·711**					
Loneliness (3-ILS total score)	**0·566**					
Sleep quality (PSQI total score)	0·482					
Diastolic blood presure		**0·877**				
Systolic blood pressure		**0·845**				
BMI			**0·869**			
Waist-hip-ratio			**0·704**	0·301		
Subjective fitness assessment	0·451		**0·576**			
Objective hearing impairment (WVT Score)				**0·805**		
Subjective hearing impairment (MAT total score)				**0·765**		
Physical activity (PASE total score)					**0·742**	
Social network size (LSNS-6 total score)				0·369	**0·643**	
Adherence to mediterranean diet (MEDAS total score)					**0·536**	
Frequency of alcohol consumption						**0·765**
Number of cigarettes per day						**0·710**

Note. Loadings of <0.30 have been omitted from the table for simplicity. Values with a loading of 0.50 or greater are highlighted. BRS Brief Resilience Scale; BMI Body Mass Index; HADS-A Hospital Anxiety and Depression Scale – Anxiety; HADS-D Hospital Anxiety and Depression Scale – Depression; LSNS-6 Lubben Social Network Scale; MAT Mini-Audio-Test; MEDAS Mediterranean Diet Adherence Screener; PASE Physical Activity Scale for the Elderly; PSS Perceived Stress Scale; 3-ILS Three item loneliness scale; WHR Waist-Hip-Ratio WVT Whispered Voice Test.

#### 3.2.3 Distribution of PC composite scores

Of all participants, 88 % exhibited an elevated risk in at least one PC composite score, as indicated by their categorisation into the highest tertile. 27 % showed high risk in only one PC, 33 % showed high risk in two PCs, and 28 % showed high risk in three or more PCs. A higher number of elevated risk domains was associated with higher age (*r* = 0·234, *p*=·003) and male gender (*t*=−2·326, *p* = 0·021), but not with APOE carrier status (*p*=·669).

**Fig. 2:**
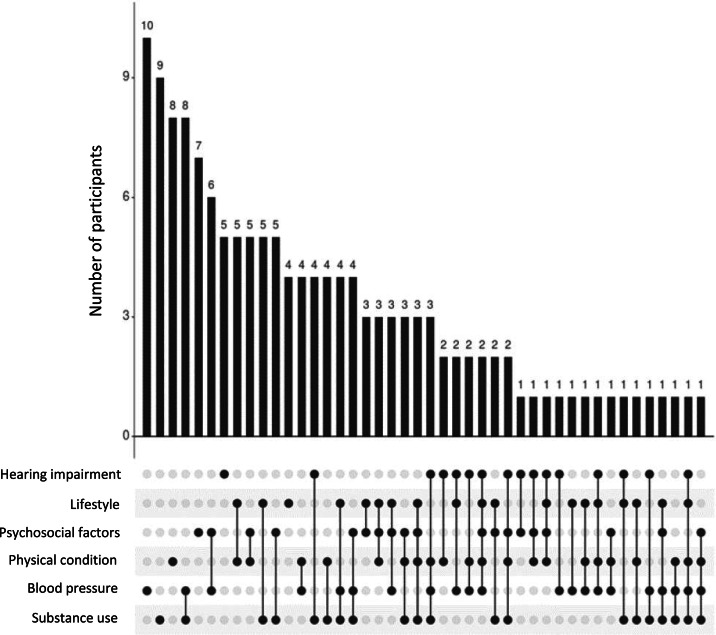
Distribution of risk factors based on principal components. UpSet plot visualising the frequency of participants with unique combinations of increased risk in each principal component (PC) composite score, highlighting that the presence of increased risk in one or two PC scores was most commonly observed in this sample. Note. Categorisation into risk groups was based on a tertile split, where the upper tertile indicated increased risk. *n* = 20 participants were not classified as increased risk for any principal component score. Non-existent intersections are omitted in the plot.

We observed the largest overlap between individuals with high scores in the blood pressure PC composite score and the substance use PC composite score (*n* = 7), followed by an overlap between those with high scores in the blood pressure PC composite score and the psychosocial factors PC composite score (*n* = 6). Co-occurrence of increased risk in any other PC composite scores was most common in individuals with increased risk in the substance use PC composite score. The distribution of all combinations is shown in Fig. 2.

### 3.3 Associations of PC composite scores with cognition, AD plasma biomarkers, and APOE genotype

#### 3.3.1 Correlation of PC composite scores with cognition

The CERAD Chandler global score correlated negatively with the hearing impairment PC composite score (*r*=−0·321, *p* < 0·001), indicating lower global cognitive performance with greater hearing impairment. The memory score correlated positively with the substance use PC composite score (*r* = 0·197, *p*=·012), indicating an association of better memory performance with lower alcohol and tobacco consumption. No other significant correlations were observed. After applying Bonferroni correction, the correlation between the Chandler score and the hearing impairment PC composite score remained significant (adjusted α′=0·0083).

#### 3.3.2 Correlation of PC composite scores with AD biomarkers and APOE genotype

The hearing impairment PC composite score, with higher scores indicating more hearing impairment, correlated positively with p-tau181 (*r* = 0·161, *p* = 0·041) and with NFL (*r* = 0·310, *p* < 0·001). The physical condition PC composite score correlated negatively with GFAP (*r*=−0·172, *p* = 0·03) and with NFL (*r*=−0·226, *p* = 0·004), indicating that a higher risk score in physical aspects was associated with lower GFAP and NFL. The substance use PC composite score correlated negatively with Aß42/Aß40 (*r*=−0·207, *p* = 0·009), indicating an association of a lower Aß42/Aß40 ratio with higher alcohol and tobacco use. After correcting for age, sex, APOE carrier status and multiple testing, only the negative correlation between the physical condition PC composite score and NFL (*r*=−0·31, *p*<·001) remained significant (adjusted α′=0·0071). To gain more nuanced insights into potential age-related effects, the analysis was repeated after dividing the sample into younger age (50–64) and older age groups (65–86), based on a median split. After correcting for sex, APOE-4-carrier status, and multiple testing, the negative association between the physical condition PC composite score and NFL was stronger in older individuals (*r*=−0·327, *p* = 0·005) compared to younger individuals (*r=*−0·207, *p* = 0·067). In addition, increased psychosocial burden was correlated with GFAP only in the group of older individuals (*r*=−0·379, *p* = 0·001).

There were no group differences in PC composite scores between APOE-4 carriers and non-carriers.

### 3.4 Lifestyle changes following the study

After six months, a subsample of *n* = 114 participants filled out a questionnaire on potential lifestyle changes since their participation. The majority of 69 participants (60.5 %) indicated that they had implemented lifestyle changes to reduce their dementia risk. Among the most frequent changes were dietary changes (58.0 %), increased physical activity (34.8 %), and stress reduction (29.0 %). Reasons for not changing the lifestyle included already having a healthy lifestyle and not seeing a reason to change (42.2 %), and a lack of resources for lifestyle changes (17.8 %).

## 4 Discussion

This study aimed to explore the prevalence of dementia risk factors among volunteers in a local prevention registry, while also gaining experiences with an individualised, single-visit risk factor assessment and counselling approach as a preparatory step towards establishing a dBHS. Participation interest was high, as the full sample was recruited after a single email announcement. Moreover, the study demonstrated the general feasibility of the program.

The most frequently identified individual risk factors were overweight/obesity, non-beneficial diet, low subjective sleep quality, stress, and hearing impairment. Principal component analyses led to the identification of six principal components: (1) psychosocial factors, (2) blood pressure, (3) physical condition, (4) hearing impairment, (5) lifestyle, and (6) substance use. Approximately 80 % of the participants showed high risk in one to three PC composite scores with high variability between PC combinations, underscoring the relevance of addressing multiple risk domains simultaneously in an individualised approach. In addition, higher age and male gender were associated with an increased number of elevated risk domains in our sample, indicating that this group might be a particularly relevant target for dBHS.

In this study, potential associations between risk factors and cognition, as well as genetic or biological markers, were examined exploratively. We observed a correlation between hearing impairment and lower cognitive performance, which is consistent with previous reports on the association of hearing impairment and MCI [23]. This finding is particularly relevant in light of evidence suggesting that hearing aids and auditory training may have a positive effect on cognition [24,25].

Regarding plasma biomarkers, we observed a negative correlation between substance use and Aß42/Aß40. However, given the low prevalence of substance use in our sample and the fact that this association did not withstand adjustment for covariates and multiple testing, this result should be interpreted with caution. A recent review emphasised that evidence on the association between AD plasma biomarkers and alcohol or tobacco consumption remains scarce, and no conclusion can currently be drawn [26]. Furthermore, we observed a negative correlation between the physical condition PC and NFL. This aligns with previous studies reporting lower GFAP and NFL concentrations in individuals with higher BMI, both in a mixed sample of cognitively unimpaired individuals and individuals with MCI or dementia [27]. It should also be noted that the role of weight may vary across the lifespan, as obesity may modulate dementia risk in midlife, whereas underweight may be an indicator of increased risk of dementia or reflect prodromal changes, which has important implications for the design of prevention interventions [28].

In our sample, there was no difference in risk profiles depending on APOE-4-carrier status. Previous studies have linked the presence of the APOE-4 allele to an increased vulnerability to lifestyle risk of AD and dementia [29,30], suggesting that APOE-4-carriers may be more likely to benefit from prevention interventions. While the mechanisms of how APOE-4 affects the human brain are not yet fully understood, there is increasing evidence that APOE-4 alters lipid and cholesterol metabolism [31,32]. No information on cholesterol levels was available in our sample, which might explain the lack of association between dementia risk factors and APOE-4-carrier status.

While these exploratory associations in our study should be interpreted with caution, they provide first insights into possible links between dementia risk factors and biological markers in this specific population of highly educated individuals who are particularly motivated in dementia prevention. Beyond these preliminary associations, the practical implications of our findings lie in the identification of clusters of modifiable risk constellations that can inform intervention prioritisation in real-world dBHS settings. Rather than addressing single risk factors in isolation, the PCA allows for the characterisation of risk constellations that may guide tailored intervention strategies. Out of the six PCs we identified, hypertension and hearing impairment are typically managed by primary care physicians or specialists. The substance use PC requires a specific program for the reduction of alcohol and tobacco consumption, although critical substance use was rare in our sample. In contrast, three components emerged as particularly actionable within dBHS: psychosocial, physical condition, and lifestyle. The psychosocial PC may be best addressed through psychological/psychotherapeutic intervention. The physical condition PC could benefit from structured weight management programs, whereas the lifestyle PC may require a combination of educational and motivational interventions. Taken together, these findings support the potential benefit of a collaborative approach in which PCA-derived profiles help to define interdisciplinary responsibilities between dBHS and medical services, while enabling dBHS to focus on the risk domains that emerged as particularly relevant targets for an individually tailored risk reduction program in our target population. Based on these results, we have already implemented structured stress-reduction courses, including progressive muscle relaxation, to target the psychosocial domain, as well as psychoeducational programs addressing lifestyle-related risk factors.

The implications of the results likely apply in particular to populations similar to ours, which consist of highly educated and prevention-motivated individuals with a strong interest in dBHS. In such an approach, participants could receive one or more of these interventions depending on their individual risk factor profile, complemented by medical treatment of hypertension and hearing impairment where appropriate.

Our study has several limitations. First, the sample size was relatively small and consisted of a specific population of highly educated individuals with a particular interest in dementia prevention. Therefore, the results are not representative of the general population. In more diverse populations, including individuals with different ethnic backgrounds or lower educational attainment, the distribution of risk factors may differ, and the focus of interventions would likely need to be adjusted accordingly. Second, the cross-sectional design does not allow conclusions to be drawn about causality or predictive validity of the identified risk factors with regard to developing dementia. However, the present study may serve as a feasibility study for future cross-sectional or longitudinal risk assessment efforts at dBHS. Third, several risk factors were assessed using subjective ratings and participant reports rather than objective measures, mainly due to the single-visit approach. Nevertheless, our selection of assessments consisted of well-established questionnaires and was guided by the recommendation of the dBHS working group [5,33]. Fourth, the correlations reported here are highly exploratory and should not be generalised. Effect sizes were small, which may partly be explained by the design and scope of this population-based pilot study. Fifth, we did not account for potential variations in the impact of risk factors across different age ranges. As the literature suggests that risk factors impact dementia risk in different ways depending on the stage of life at which they occur, a more fine-grained assessment of risk factors in relation to age could provide valuable insights for more individualised risk estimations. Finally, the counselling provided in this study was not systematically evaluated or followed up, which means we cannot conclude the effects, acceptance, or understanding of the communicated information. This aspect will be addressed in future studies and the refinement of the program. In this context, the ongoing PreTAD study explores the needs and anticipated effects of hypothetical risk communication in cognitively healthy individuals at increased risk of dementia [34]. In addition, a brain health service for risk assessment and risk communication is currently being implemented at the Cologne Alzheimer’s Prevention Center, which builds on the results and experiences of the current trial. Therefore, we expect to increase the available data on risk factors and direct greater attention towards the evaluation of risk communication and its effects.

In conclusion, this study demonstrates the high level of interest among participants in an individualised dementia risk assessment within a dBHS framework and highlights the feasibility of a single-visit approach. By identifying common and modifiable risk factors in this population, particularly in areas such as psychosocial factors, physical condition, and lifestyle, we have established a foundation for developing individually tailored risk reduction intervention programs. The follow-up assessment on lifestyle changes showed that the majority of participants implemented lifestyle changes following the risk assessment and communication. These findings provide initial insights into which risk factors may be most relevant and actionable for populations likely to seek dBHS. While further research in more diverse populations is needed to increase generalisability, this approach provides a pragmatic and practical model for individualised dementia prevention, contributing to a broader understanding of risk factor distribution and aligning with global approaches for promoting brain health.

## Ethics approval and consent to participate

The study was approved by the ethical committee of the Medical Faculty of the University of Cologne (ID 20–1096). All participants gave written informed consent prior to the study. The protocol was registered in the German Clinical Study Register (Deutsches Register Klinischer Studien, DRKS, ID DRKS00020767).

## Consent for publication

Not applicable.

## Availability of data and materials

The data that support this manuscript are not publicly available, but may be provided upon reasonable request.

## Funding sources

The study was funded by research funds of the Medical Faculty and the University Hospital Cologne, 10.13039/501100009983University of Cologne. In addition, the study has received funding from the non-profit association Kölner Verein für seelische Gesundheit e.V.

## Declaration of generative AI and AI-assisted technologies in the manuscript preparation process

Nothing to disclose.

## CRediT authorship contribution statement

**Lena Sannemann:** Writing – original draft, Visualization, Investigation, Formal analysis, Data curation, Conceptualization. **Michelle Gerards:** Writing – original draft, Visualization, Investigation, Formal analysis, Data curation. **Lara Bohr:** Writing – review & editing, Investigation. **Frederic Brosseron:** Formal analysis. **Claus Escher:** Writing – review & editing, Investigation, Conceptualization. **Franziska Kalthegener:** Writing – review & editing, Investigation, Formal analysis, Data curation. **Theresa Müller:** Writing – review & editing, Investigation, Conceptualization. **Alfredo Ramírez:** Formal analysis. **Philip Zeyen:** Writing – review & editing, Investigation, Formal analysis, Data curation. **Frank Jessen:** Writing – review & editing, Supervision, Funding acquisition, Conceptualization. **Ayda Rostamzadeh:** Writing – review & editing, Supervision, Conceptualization.

## Declaration of competing interests

Frank Jessen reports was provided by University of Cologne; he has received personal fees for funding and advisory services from the following companies: AC Immune, Biogen, Danone/Nutricia, Eisai, GE Healthcare, Grifols, Janssen, Lilly, MSD, Novo Nordisk, and Roche. Ayda Rostamzadeh reports a relationship with Eisai Europe Ltd that includes: speaking and lecture fees. Ayda Rostamzadeh reports a relationship with Eli Lilly and Company that includes: speaking and lecture fees. Ayda Rostamzadeh is a Member of the European Brain Initiative. Philip Zeyen reports a relationship with Novo Nordisk Inc that includes speaking and lecture fees. The other authors declare that they have no known competing financial interests or personal relationships that could have appeared to influence the work reported in this paper.

## Appendix Supplementary materials

Image, application 1
