# Supplementary material for: Dementia risk factor assessment in a local Alzheimer’s prevention population: a German cross-sectional, observational study
Source: J Prev Alzheimers Dis. 2026 Apr 3;13(6):100556. doi: 10.1016/j.tjpad.2026.100556 (PMC13088994; doi:10.1016/j.tjpad.2026.100556)
Supplement: Supplementary file 1 [file mmc1.docx]

**Supplementary material**

**Table of Contents**

[S1. Assessment of demographic information and medical history 2](#_Toc207287867)

[Table S1. Assessment methods of lifestyle risk factors, risk factors of physical and mental health, and cognitive performance. 2](#_Toc207287868)

# **S1. Assessment of demographic information and medical history**

The following demographic information was assessed with a self-developed questionnaire: year of birth, sex, mother tongue, proficiency in German language, highest level of education (school), highest level of professional training, employment status, retirement status, living situation, family history of dementia.

A detailed medical history was taken for the following conditions: hypercholesterolemia, thyroid disease, hypertension, diabetes mellitus type 2, traumatic brain injury, kidney disease, coronary heart disease, atrial fibrillation, stroke, Parkinson's syndrome, vitamin deficiency, depression, anxiety, drug addiction, hearing impairment, visual impairment, olfactory impairment, other diseases (freeform).

The history of medication intake was recorded for the following medications: antidepressant medication, proton-pump inhibitors ≥4 weeks, hormone replacement therapy, opioids ≥4 weeks, benzodiazepines ≥4 weeks, other medication (freeform).

# **Table S1. Assessment methods of lifestyle risk factors, risk factors of physical and mental health, and cognitive performance.**

|  | **Risk Factor** | **Method of Assessment** | **Presence of Risk Factor** |
| --- | --- | --- | --- |
| Lifestyle risk factors | Adherence to Mediterranean diet | MEDAS ^1^ | Score ≤ 7 |
|  | Physical activity | PASE ^2^ | Score ≤ 110 |
|  | Size of social network | LSNS-6 ^3^ | Score < 12 |
|  | Alcohol consumption | Number of standard drinks per week | Standard drinks/week >7 (women); > 14 (men) |
|  | Tobacco consumption | Period of tobacco consumption, number of cigarettes/day | Current occasional or daily tobacco consumption |
|  | Sports with a high risk of head injury | Period of risk sports | Past or present risk sports |
| Physical and mental health | Quality of sleep | PSQI ^4^ | Score ≥ 6 |
|  | Stress | PSS-10 ^5^ | Women: <60Y: Score ≥ 22·71; ≥60Y: Score ≥ 21·94  Men: <60Y: Score ≥ 22·14; Men ≥60Y: Score ≥ 20·01 |
|  | Resilience | BRS ^6^ | Score < 3 |
|  | Loneliness | 3-ILS ^7^ | Score ≥ 6 |
|  | Anxiety (current symptoms) | HADS-D ^8^ | Score ≥ 8 |
|  | Depression (current symptoms) | HADS-D ^8^; GDS ^9^ | HADS-D: Score ≥ 8; GDS: Score ≥ 6 |
|  | Subjective hearing deficits | MAT ^10^ | <60Y: Score: ≥ 2; ≥60Y: Score ≥ 3 |
|  | Risk of parodontitis | Parodontitis Riskscore ^11^ | Score ≥ 11 |
|  | Subjective evaluation of physical fitness | Self-developed item [Likert-Scale, 0-4] | N/A |
| Physical exam | Abdominal obesity | Waist-hip ratio ^12^ | Women ≥0·80; men ≥0·90 |
|  | Hypertension | Blood pressure measurement onsite ^13^ | Systolic blood pressure ≥140 mmHg |
|  | Body Mass Index (BMI) | Measurement onsite | Overweight: 25-29·9 kg/m^2^, Obesity: ≥30 kg/m^2^ |
|  | Objective hearing impairment | Whispered Voice Test ^14^ | Hearing impairment (Score <1) in one or both ears |
| Neuropsychology | Cognitive performance | CERAD+ ^1516^(16)(10)(9) | MCI defined as < -1·5 SD in any test, < -1 SD in two tests of one domain, or <-1 SD in three tests of different domains |
|  | Subjective cognitive decline (SCD) | SCD-Interview ^17^ | SCD in any cognitive domain (memory, language, attention, executive function, other) |
|  | Resources and self-management skills | FERUS ^18^ | N/A |
| *MEDAS*, Mediterranean Diet Adherence Screener; *PASE*, Physical Activity Scale for the Elderly; *PSQI*, Pittsburgh Sleep Quality Index; *PSS-10*, Perceived Stress Scale; *BRS*, Brief Resilience Scale; *LSNS-6*, Lubben Social Network Scale; *3-ILS*, Three item loneliness scale; *HADS-D*, Hospital Anxiety; and Depression Scale; *GDS*, Geriatric Depression Scale; *MAT*, Mini-Audio-Test; *CERAD+,* Consortium to Establish a Registry for Alzheimer's Disease battery plus version; *FERUS*, Questionnaire to assess resources and self-management skills [Fragebogen zur Erfassung von Ressourcen und Selbstmanagementfähigkeiten]. | | | |

**References**

1. Hebestreit K, Yahiaoui-Doktor M, Engel C, et al. Validation of the German version of the Mediterranean Diet Adherence Screener (MEDAS) questionnaire. *BMC Cancer* 2017; 17: 341.

2. Washburn RA, Smith KW, Jette AM, et al. The Physical Activity Scale for the Elderly (PASE): development and evaluation. *J Clin Epidemiol* 1993; 46: 153–162.

3. Lubben J, Blozik E, Gillmann G, et al. Performance of an abbreviated version of the Lubben Social Network Scale among three European community-dwelling older adult populations. *Gerontologist* 2006; 46: 503–513.

4. Buysse DJ, Reynolds CF, Monk TH, et al. The Pittsburgh Sleep Quality Index: a new instrument for psychiatric practice and research. *Psychiatry Res* 1989; 28: 193–213.

5. Klein EM, Brähler E, Dreier M, et al. The German version of the Perceived Stress Scale - psychometric characteristics in a representative German community sample. *BMC Psychiatry* 2016; 16: 159.

6. Chmitorz A, Wenzel M, Stieglitz R-D, et al. Population-based validation of a German version of the Brief Resilience Scale. *PLoS One* 2018; 13: e0192761.

7. Spitzer M. Einsamkeit – erblich, ansteckend, tödlich. *Nervenheilkunde* 2016; 35: 734–741.

8. Zigmond AS and Snaith RP. The hospital anxiety and depression scale. *Acta Psychiatr Scand* 1983; 67: 361–370.

9. Yesavage JA and Sheikh JI. 9/Geriatric Depression Scale (GDS). *Clinical Gerontologist* 1986; 5: 165–173.

10. Löhler J, Walther LE and Schlattmann P. Der Mini-Audio-Test (MAT). Screening-Fragebogen zur Ermittlung einer relevanten Schwerhörigkeit ab dem 50. Lebensjahr. *Laryngorhinootologie* 2013; 92: 815–822.

11. Deutsche Gesellschaft für Parodontologie e.V. Der Parodontitis Risikoscore-ein Selbsttest. *Ratgeber Parodontitis verstehen, vermeiden und behandeln*.

12. WHO. (2011) Waist Circumference and Waist-Hip Ratio: Report of a WHO Expert Consultation, http://www.who.int/entity/nutrition/publications/obesity/WHO_report_waistcircumference_and_ waisthip_ratio/en/ (Geneva, 2008., accessed 18 November 2024).

13. Williams B, Mancia G, Spiering W, et al. 2018 ESC/ESH Guidelines for the management of arterial hypertension. *Eur Heart J* 2018; 39: 3021–3104, https://academic.oup.com/eurheartj/article/39/33/3021/5079119 (2018).

14. Swan IR and Browning GG. The whispered voice as a screening test for hearing impairment. *J R Coll Gen Pract* 1985; 35: 197.

15. Morris JC, Heyman A, Mohs RC, et al. The Consortium to Establish a Registry for Alzheimer's Disease (CERAD). Part I. Clinical and neuropsychological assessment of Alzheimer's disease. *Neurology* 1989; 39: 1159–1165, https://pubmed.ncbi.nlm.nih.gov/2771064/ (1989).

16. Chandler MJ, Lacritz LH, Hynan LS, et al. A total score for the CERAD neuropsychological battery. *Neurology* 2005; 65: 102–106.

17. Miebach L, Wolfsgruber S, Polcher A, et al. Which features of subjective cognitive decline are related to amyloid pathology? Findings from the DELCODE study. *Alzheimers Res Ther* 2019; 11: 66.

18. Jack M. Fragebogen zur Erfassung von Ressourcen und Selbstmanagementfähigkeiten. FERUS. *Hogrefe,* 2007.
